# Effect of Beetroot Juice Supplementation on Muscle Soreness and Performance Recovery after Exercise-Induced Muscle Damage in Female Volleyball Players

**DOI:** 10.3390/nu15173763

**Published:** 2023-08-28

**Authors:** Mohammad Hemmatinafar, Leila Zaremoayedi, Maryam Koushkie Jahromi, Stacey Alvarez-Alvarado, Alexei Wong, Alireza Niknam, Katsuhiko Suzuki, Babak Imanian, Reza Bagheri

**Affiliations:** 1 Department of Sport Science, Faculty of Education and Psychology, Shiraz University, Shiraz 71946-84334, Iran; 2 Department of Neurology, College of Medicine—Jacksonville, University of Florida, Jacksonville, FL 32209, USA; 3 Department of Health and Human Performance, Marymount University, Arlington, TX 22207, USA; 4 Faculty of Sport Sciences, Waseda University, 2-579-15 Mikajima, Tokorozawa 359-1192, Japan; 5 Department of Exercise Physiology, University of Isfahan, Isfahan 81746-73441, Iran

**Keywords:** beetroot juice, muscle soreness, recovery, female athletes, volleyball

## Abstract

Background: Beetroot juice (BRJ) contains various bioactive compounds suggested to be effective in improving athlete recovery. However, the number of studies evaluating the effects of BRJ on recovery and muscle soreness (MS) indicators in female athletes is limited. Therefore, the present study aimed to determine the effects of BRJ consumption on the performance recovery indicators and MS after exercise-induced muscle damage (EIMD) in female volleyball players. Methods: Twelve young female volleyball players were evaluated in this study. We utilized a randomized, cross-over, and double-blind design during two phases with a 30-day interval (wash-out). During each phase, EIMD was performed first, followed by BRJ or placebo (PLA) supplementation for two days (eight servings of 50 mL). Recovery monitoring of performance indicators and MS was performed after EIMD. The results of wall-sit, V sit and reach (VSFT), vertical jump height (VJH), pressure pain threshold (PPT), and thigh swelling (Sw-T) tests were recorded 48 h after EIMD. Also, the Perceived Muscle Soreness was recorded using the visual analog scale (VAS) 12 (MS-12 h), 24 (MS-24 h), and 48 (MS-48 h) hours after EIMD. Results: The data were analyzed using two-way repeated measures of ANOVA at *p* < 0.05. Compared to PLA, BRJ supplementation improves wall-sit performance after EIMD (*p* < 0.05), while reducing Sw-T and perceived muscle soreness (*p* < 0.05). However, no significant difference was observed between PLA and BRJ in VJH and VSFT performance after EIMD (*p* > 0.05). Conclusions: Our findings indicate that the consumption of BRJ in female volleyball players can be useful for improving some recovery indicators, such as muscle endurance, perceived muscle soreness, and tissue edema, after EIMD.

## 1. Introduction

Volleyball is one of the world’s most popular sports, requiring high physical abilities at professional levels [1,2]. Successful volleyball performance requires intense movement tasks, such as jumps, rapid direction changes, and dives, alternately with intervals of low-intensity activities [1,3]. Due to the nature of these motor tasks, eccentric muscle contractions seem to play an essential role in volleyball techniques and skills [1,4]. This is important to point out since eccentric contractions can contribute to muscle micro-damages and muscle soreness after exercise [1,5]. On the other hand, in many tournaments, the interval between volleyball matches is often less than two or three days, which can increase the risk of insufficient recovery and excessive stress for volleyball players [1]. Therefore, accelerating recovery and using preventative and practical strategies to reduce muscle soreness are valuable elements for these athletes [1].

One effective strategy for athlete recovery that has attracted the attention of many researchers is the short-term use of nutritional supplements [6]. For instance, beetroot juice (BRJ) is a nutritional supplement containing high amounts of nitrates, which are claimed to improve physical performance via increased nitric oxide (NO) [7,8]. The suggested BRJ supplementation should be ~2.5 h before exercise, as nitrate levels peak between 2 and 3 h after consumption [9]. In addition to nitrate, BRJ also contains other compounds, such as betalain, flavonoids, and phenolic compounds, which can affect the biological function of this supplement [10]. For example, betalain and plant flavonoids have been shown to have potential antioxidant and anti-inflammatory effects [11].

Concerning sports performance, studies have shown that BRJ supplementation can improve endurance performance, increase muscle blood flow [7], enhance oxygen transfer to mitochondria, and improve muscle contraction–relaxation processes [12,13]. However, its effect on power performance is less studied. Some recent findings have supported the impact of nitrate-rich supplements on anaerobic performance [14]. Interestingly, the observed benefits of BRJ seem to affect only type II muscle fibers [15]. In these fibers, NO stimulates the release of calcium into the sarcoplasm, through the involvement of calsequestrin [8], reduces the rate of phosphocreatine degradation, and reduces the cost of adenosine triphosphate (ATP) at ranges of exercise intensity [16]. The consequence of these intracellular mechanisms is to improve performance and reduce fatigue during high and maximal-intensity activities [17]. Indeed, prior research showed that acute BRJ supplementation increases the peak power during the Wingate test [18,19].

In addition to these ergogenic effects, BRJ supplementation seems useful in performance recovery and reduction in muscle pain [11]. The elevated concentrations of nitrate in BJR can be converted into NO, a compound that appears essential in regenerating damaged skeletal muscles [11,20]. Moreover, NO has been suggested to reduce the muscle damage caused by inflammatory cells by increasing their apoptosis [8]. NO also reduces the lysis of muscle cells (caused by the action of neutrophils) and the concentration of superoxide, resulting in a reduced formation of reactive radical mediators [21]. Therefore, BRJ supplementation has been suggested to enhance recovery from delayed onset muscle soreness due to its composition and consequent effects on muscle function, injury, and repair processes [11]. However, research to date has produced conflicting results. In this regard, two investigations by Clifford et al. [22,23] showed improved recovery in the countermovement jump (CMJ) height and reactive strength index (RSI) after BRJ supplementation post-exercise. These studies demonstrated no beneficial effect of BRJ supplementation on maximal isometric voluntary contractions (MIVC) [22,23]. Another study also reported that BRJ supplementation did not affect muscle soreness, CMJ height, or knee extensor MIVC after intense endurance activity [24]. On the other hand, a review by Wickham and Spriet (2019) summarized the potential sex-based differences in the effects of nitrate supplementation on performance and showed that, despite significant anthropometric, metabolic, and physiological differences between the sexes, it is abundantly clear that women are underrepresented in BRJ supplementation research [25]. Three prior studies, that investigated cycling performance outcomes in females after BRJ supplementation, found no significant effects in the evaluated parameters [26,27,28]. Yet, two other studies found positive performance results in female swimmers [29] and kayakers [30]. These studies have investigated the effect of BRJ on cycling and swimming performance, which have different movement and biomechanics patterns than volleyball. It should be noted that the biomechanics of movements and contractions in volleyball are mostly based on a stretch-shortening cycle.

Therefore, considering the contradictory and limited evidence about the effect of BRJ on performance recovery and muscle soreness symptoms [11], especially in female volleyball players, the present study investigated the effect of oral BRJ supplementation after exercise-induced muscle damage (EIMD) on the recovery of functional parameters (endurance and flexibility of leg muscles and jumping performance) and muscle soreness symptoms (pressure pain threshold (PPT) and thigh swelling (Sw-T)).

## 2. Methods

### 2.1. Participants

Fourteen semi-professional female volleyball players with almost five years of volleyball experience, who had at least three training sessions and two resistance training sessions per week, voluntarily participated in this research. These players were at the university competition level. The demographic information of the participants is listed in Table 1. Participants had no known diseases or medical issues, no history of allergy to beets or BRJ, and were not consuming any supplements or medications. Furthermore, the participants did not smoke or consume alcoholic or caffeinated beverages at the time of data collection. Prior to the implementation of the intervention, the study procedures were explained to the participants and consent was obtained. This research was approved by the Research Institute of Sports Sciences (Iran), with the ethics identifier SSRI.REC-2203-1551, and performed in accordance with the Declaration of Helsinki.

#### Sample Size Calculation

The number of participants in this study was determined based on the study by Clifford et al. [22], according to which BRJ led to a significant improvement in recovery of a vertical jump compared to a placebo (Cohen’s d = 1.25). Using G*Power 3.1, considering the confidence interval of 95%, and the analysis power of 0.80, it was found that at least 9 participants (for each condition) are needed for this study. In order to ensure a sufficient sample size, 14 participants were selected for this study.

### 2.2. Study Design

This study was carried out in a crossover, randomized, and double-blinded manner. Before the beginning of the investigation, the participants underwent a familiarization session. During this session, participants were familiarized with all testing protocols and procedures. Moreover, before data collection, sensitivity to BRJ and PLA was tested by ingesting 50 mL of the respective supplementation. The first phase of the research took place 48 h after the supplementation sensitivity test. This phase included two sessions. The first session was composed of EIMD, while the second session included the performance of functional tests. Two of the volunteers withdrew from the study due to digestive disorders caused by BRJ supplementation. In total, 6 participants consumed 50 mL of BRJ and the other 6 participants ingested 50 mL of PLA at 2, 6, 10, 14, 26, 30, 34, and 38 h after EIMD (400 mL per two days). Functional tests were performed 48 h after EIMD. Furthermore, the perceived muscle soreness was recorded at 12 (MS-12), 24 (MS-24), and 48 (MS-48) hours after EIMD. In the second stage, EIMD, supplementation, and functional tests were performed during 2 sessions similar to the first stage (with the replacement of BRJ and PLA members). In order to reduce the influence of hormonal variations during the menstrual cycle on variables of interest, the EIMD, supplementation, and functional tests were performed during the late follicular phase (10 days after the start of menstruation). The menstrual cycle status for each individual was determined through the Menstrual Cycle Questionnaire [31]. The second phase was performed during the next follicular phase, approximately 30 days after the first session.

### 2.3. Exercise-Induced Muscle Damage Protocol

Prior to the EIMD protocol, the participants performed a 10 min warm-up composed of dynamic movements, slow running, and stretching exercises. Thereafter, participants completed 200 vertical jumps with weighted vests (equivalent to 10% of body weight) to induce muscle damage. For this purpose, each person performed 10 sets of 20 maximum jumps (one jump every 4 s). Two minutes of rest (sitting in a chair) were included between sets. To ensure the rigidity of the program, the RPE scale was presented and assessed by the participants immediately after the test. All subjects performed the EIMD at 8:00 am and 1 h post meal ingestion (100 g banana). The EIMD protocol was adapted from the previous literature on BRJ supplementation [22,32]. During the study, all participants were members of the same training camp and their diet and training protocol were the same under the supervision of trainers.

### 2.4. Supplementation Procedures

**Figure 1:**
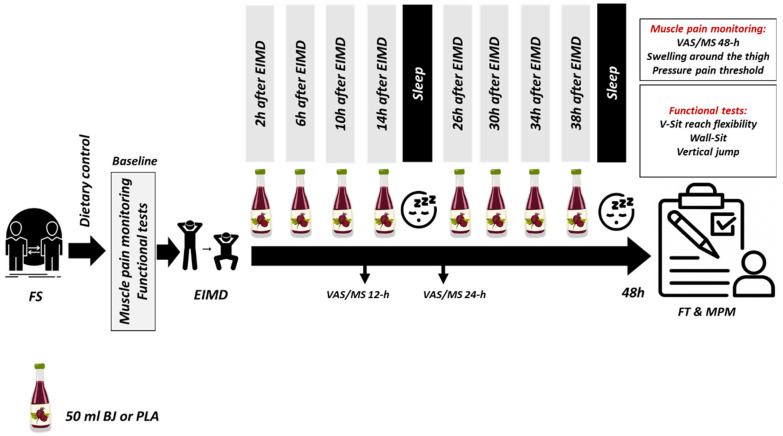
Supplementation and testing protocol. FS: Familiarization session, EIMD: Exercise-induced muscle damage, FT: Functional tests, MPM: Muscle pain monitoring, BJ: Beetroot juice, PLA: Placebo, ml: Milliliters, VAS: Visual analog scale, MS: Muscle soreness.

Participants were supplemented with BRJ (prepared from red beet in Zarghan Lepoi Farms, Shiraz, Iran) or PLA (Geernosense, Shiraz, Iran) 48 h after EIMD. Participants were required to ingest 50 mL of BRJ or PLA with each meal at 2, 6, 10, 14, 26, 30, 34, and 38 h after EIMD (400 mL per two days) (Figure 1). The details of BRJ and PLA drinks are listed in Table 2. Since the drinks could not be matched for taste, the objectives of the study were concealed from the participants. The participants were unaware that BRJ was under investigation and of the antioxidant nature of both types of drinks. This protocol was previously implemented by Clifford et al. (2017) [32]. The participants were provided with eight sealed bottles containing 50 mL of BRJ or PLA in each research stage. Specific instructions for use and timing were provided on all the bottles.

### 2.5. Muscle Pain Monitoring

Muscle soreness was measured using a visual analog scale (VAS) scale. A 10 cm linear image was provided on paper to the participants. “No pain” was written on one end of the line, while “maximum pain” was portrayed on the opposite end. Participants determined their perception of the intensity of muscle soreness with a cross mark “x” on the lines before, 12, 24, and 48 h after EIMD. The amount of muscle soreness for each participant was recorded in millimeters with a ruler by measuring the distance from the origin of the line to the marked “x” [6]. The amount of swelling and pressure pain threshold in the middle part of the femur was also recorded before (baseline) and 48 h after EIMD in fasting conditions (10 h). To determine the midpoint of the femur, the greater trochanter of the femur and the tibial prominence of the dominant leg was marked while the participant was standing. The perimeter of the femur was measured three times (without creating folds in the skin) to determine the amount of swelling around the thigh (Sw-T), using a tape measure to the nearest 1 mm. The mean values were recorded as the swelling score around the femur. The pressure pain threshold (PPT) amount was also determined at the femur midpoint using a mercury pressure cuff. The subjects sat on a chair (knee in 90-degree flexion), and a plastic tube (diameter of 2.5 cm and length of 25 cm) was placed around the thigh (femur midline) of the dominant leg. The mercury pressure gauge cuff was placed around the participant’s thigh, followed by uniform inflation. The investigator recorded the pressure level at the moment of pain onset as the PPT in mmHg.

### 2.6. Functional Tests

Following the muscle pain monitoring, functional tests were performed in the following order: V-Sit and reach flexibility test (VSFT), vertical jump height (VJH), and wall-sit. During the VSFT, participants sat shoeless on the floor with their soles 30 cm apart. A meterstick was placed between the participant’s legs, so the 23 cm mark aligned with the participant’s heels [33]. Subjects were later asked to place both hands together and extend forward as far as possible. The best of three attempts was recorded as the final score. The participants performed the VJH test on the Ergo jump screen (Desikala, Iran) three times (1 min rest between each time) utilizing standard procedures. The best jump was recorded as the final score [34]. The wall-sit test was used to evaluate muscle endurance. During this test, subjects were instructed to position their feet at shoulder width and lean against the wall. Then, subjects were asked to adopt the squat position by lowering the center of gravity and bending the knee and femur until their femurs were parallel to the ground (hip and knees in 90-degree flexion) [35]. The time the subjects kept their position was recorded with a stopwatch. It should be noted that functional tests were also performed at baseline and 48 h after EIMD.

### 2.7. Data Analysis

The collected data were analyzed using SPSS software (version 26, IBM-SPSS Inc., Chicago, IL, USA). Two-way (Condition × Time) repeated measures of ANOVA and Bonferroni post hoc tests were used to analyze the collected data. The significance level was set to *p* < 0.05.

## 3. Results

**Figure 2:**
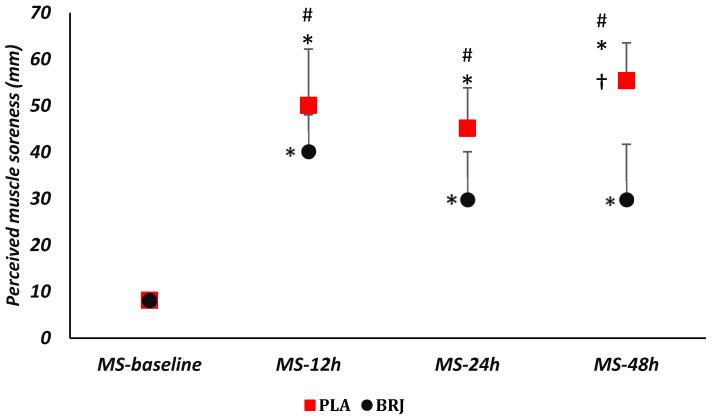
Perceived muscle soreness changes using the VAS scale in PLA or BRJ conditions. *: Significant difference with MS-baseline (*p* < 0.05). †: Significant difference with MS-24 h (*p* < 0.05). #: Significant difference between PLA and BRJ conditions (*p* < 0.05). BRJ: Beetroot juice, PLA: Placebo.

The two-way repeated measure of ANOVA showed that the main effect of the condition [F = 17.6, *p* = 0.001, η^2^ = 0.6], time [F = 143.6, *p* = 0.000, η^2^ = 0.9], and interaction (condition × time) [F = 12.6, *p* = 0.000, η^2^ = 0.5] was significant for perceived muscle soreness. The post hoc test showed that MS-12 h, MS-24 h, and M-48 h have significantly increased compared to MS-baseline in both PLA and BRG (*p* < 0.05). No significant difference was observed between MS-12 and MS-48 and between MS-12 and MS-24 after PLA or BRJ (*p* > 0.05). However, MS-48 showed a significant increase after PLA compared to MS-24 (MD = 10.25, *p* = 0.003), while no significant changes were observed after BRJ (*p* > 0.05). In addition, MS-baseline was not significantly different between PLA and BRJ (*p* > 0.05), however, MS-12, MS-24, and MS-48 were significantly increased after PLA compared to BRJ (*p* < 0.05) (Figure 2, Table 3).

**Figure 3:**
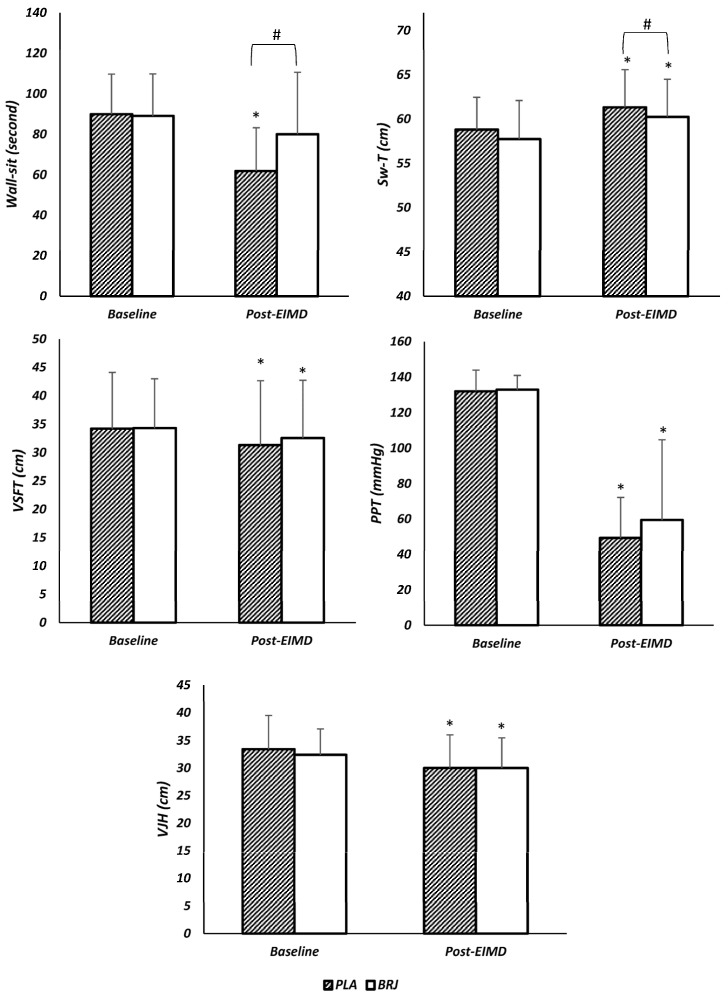
Changes in functional parameters of PLA and BRJ. Sw-T: Swelling around the thigh, PPT: pressure pain threshold, VSFT: V-sit reach flexibility test, VJH: Vertical jump height. Post-EIMD: 48 h after exercise-induced muscle damage #: significant difference in BRJ compared to PLA conditions (*p* < 0.05). *: Significant difference with baseline (*p* < 0.05).

The results showed that the main effect of condition was not significant for wall-sit performance [F = 4.2, *p* = 0.06, η^2^ = 0.3], while the main effect of time [F = 66, *p* = 0.000, η^2^ = 0.9] and interaction (conditions x time) were significant [F = 17, *p* = 0.002, η^2^ = 0.6]. The post hoc test showed that, 48 h after EIMD compared to baseline, the performance of wall-sit in PLA decreased significantly (*p* < 0.05), while no significant changes were observed after BRJ (*p* > 0.05). Wall-sit performance at baseline was not significantly different between PLA and BRJ while, 48 h after EIMD, Wall-sit performance in BRG conditions was significantly improved compared to PLA (*p* < 0.05) (Table 3, Figure 3).

In addition, the main effect of time [F = 58.9, *p* = 0.000, η^2^ = 0.8] and condition [F = 11.99, *p* = 0.005, η^2^ = 0.5] was significant for Sw-T, while no significant interaction was observed for Sw-T [F = 0.001, *p* = 0.999, η^2^ = 0.001]. Th post hoc test showed that, 48 h after EIMD compared to baseline, Sw-T increased significantly in PLA and BRJ (*p* < 0.05). Also, the results showed that there was no significant difference between Sw-T in baseline between PLA and BRJ (*p* > 0.05), while 48 h after EAMD, Sw-T was significantly reduced in BRJ compared to PLA (*p* < 0.05) (Table 3, Figure 3).

The main effect of time was significant for PPT [F = 79.4, *p* = 0.000, η^2^ = 0.9], VSFT [F = 20.7, *p* = 0.001, η^2^ = 0.6], and VJH [F = 68.4, *p* = 0.000, η^2^ = 0.9], but the main effect of condition was not significant for PPT [F = 1.6, *p* = 0.23, η^2^ = 0.12], VSFT [F = 0.4, *p* = 0.51, η^2^ = 0.04], and VJH [F = 0.75, *p* = 0.4, η^2^ = 0.06]. Also, no significant interaction effect was observed for PPT [F = 0.66, *p* = 0.43, η^2^ = 0.06], VSFT [F = 2.9, *p* = 0.11, η^2^ = 0.2], and VJH [F = 3.5, *p* = 0.09, η^2^ = 0.2]. The post hoc test showed that, 48 h after EIMD compared to baseline, PPT had a significant decrease in both PLA and BRJ. (*p* < 0.05). Also, 48 h after EIMD, the performance of VSFT and VJH showed a significant decrease compared to the baseline for both PLA and BRJ (*p* < 0.05) (Table 3, Figure 3).

## 4. Discussion

This study was conducted to determine the effect of BRJ supplementation after EIMD on the recovery of functional parameters (endurance and flexibility of leg muscles and vertical jump performance) and muscle soreness symptoms (PPT, Sw_T, perceived muscle soreness). The findings showed that BRJ supplementation improved muscle endurance, Sw-T, and perceived muscle soreness after EIMD in female volleyball players. However, no significant differences were observed between PLA and BRJ in PPT, VSFT, and VJH. Therefore, BRJ supplementation may have beneficial effects on some muscle recovery indicators.

In line with these findings, a prior meta-analysis showed the beneficial effects of BRJ supplementation on reducing the perception of muscle soreness (especially at 48 and 72 h after various exercises) [36]. In addition, Daab et al. (2021) showed that BRJ supplementation, compared to PLA, can reduce muscle soreness immediately and 24 h after the Loughborough intermittent sprint test (LIST) in semi-professional male soccer players [37]. Despite this, and unlike the present study, there was no significant difference in muscle soreness between PLA and BRJ at intervals of 48 and 72 h following simulated match play in soccer players [37]. Of note, during the Daab et al. (2021) study, the supplement was taken for 7 days (3 days before, the day of, and 3 days after LIST, 150 mL/twice per day) while, in the present study, the supplement was taken 48 h after the EIMD [37]. Furthermore, Daab et al. (2021) evaluated muscle soreness after the LIST protocol using a numerical scale [37] while, in the present study, we evaluated muscle soreness after the jump training protocol (200 jumps) and using the VAS scale. Other studies have also reported no significant effects of BRJ supplementation compared to PLA on reducing muscle pain [24,38]. For instance, a study on trained runners showed that BRJ supplementation within 2 days after a marathon had no significant effect on the perception of muscle soreness (48 h after exercise) [24]. The authors noted that muscle pain perception using VAS increased slightly in PLA and BRJ conditions immediately after the marathon and then returned to near baseline values (before the marathon) 24 and 48 h later [24]. One potential reason for the lack of difference between BRJ and PLA could be attributed to the participants’ fitness level. Participants in this study had completed approximately 16 marathons before the intervention, had between 7 and 13 years of running experience, and performed a similar training modality as the sports test per training records [24]. Larsen et al. (2019) showed similar results in recreationally active men and women, where BRJ supplementation did not have any significant effects on PPT or muscle soreness (using a Likert scale) after outdoor exercise [38]. Nevertheless, the training protocol, measurement tools, and participants’ sports history differed from our present study. Clifford et al. (2017) also showed BRJ increased PPT 72 h after EIMD (100 drop jumps) compared to PLA in recreationally active men [32]. However, the present study measured PPT 48 h after EIMD. Therefore, the difference in the timing of PPT monitoring is another possible reason for the discrepancy between the results of this study and previous studies [23,32]. In addition, it seems that the difference in the gender of the studied participants [23,32,37], the type of sports tests used [37], and the volume of activity (100 jumps vs. 200 jumps) [32] can make the findings inconsistent. Due to inconsistent results, more studies on the effect of BRJ on muscle soreness are needed.

The analgesic effects of BRJ supplementation have been mainly attributed to its active phytochemicals (betalain and plant polyphenols) [32]. Therefore, the reduction in MS-48h after BRJ supplementation in the present study may be caused by the anti-inflammatory and antioxidant compounds of BRJ. Despite this, some studies have shown the analgesic effects of BRJ independent of changes in inflammatory markers [11,23]. Hence, the analgesic effects of BRJ cannot be credited only to inflammatory pathways and oxidative stress markers. However, a recent meta-analysis suggested that further studies be conducted on the interaction of BRJ’s analgesic effects and muscle inflammation pathways [36]. In addition to plant polyphenols, BRJ contains large amounts of nitrates, which increase the available levels of NO in the body [36]. Based on the available evidence, NO can activate the nociceptors of C fibers and increase pain sensation [39]. Hence, increased NO may counteract the analgesic effects of polyphenols in BRJ. However, the exact role of NO or BRJ supplementation in this regard is still unclear. Moreover, NO has been suggested to activate satellite cells and increase follistatin to repair the damage caused by muscle pain [39]. Therefore, one potential reason for pain reduction and improved muscle endurance performance in the present study may be improving the repair process via NO. However, a study comparing nitrated beverages and BRJ found that BRJ was more effective than nitrated beverages for reducing muscle pain associated with EIMD, and any analgesic effects were likely due to phytonutrients in BRJ other than nitrates [32]. Additionally, recent evidence has suggested muscle soreness is an acute compression axonopathy with tissue microdamage and oxidative stress enhanced by immune-mediated inflammation [40]. According to this theory, NO plays a role in the secondary phase of micro neurological damage (i.e., inflammation and repair by the immune system) after eccentric exercise [40]. Therefore, considering the complex underlying mechanisms of muscle soreness and the lack of research evidence about the role of NO in its control, more studies should be conducted on the interaction of NO caused by BRJ and muscle soreness.

The findings showed that BRJ supplementation led to concomitant reductions in muscle soreness and increases in Wall-sit performance (muscular endurance) after EIMD (*p* < 0.05), while no significant difference in VJH and VSFT performance was established. Several studies have shown the positive effect of BRJ on performance recovery indicators such as maximal voluntary isometric strength (MVIC), vertical jump, and aerobic endurance. However, other investigations have not reported any beneficial effects [38,41]. According to the findings from a meta-analysis by Jones et al. (2021) [36], the improvements in VJH and MVIC during recovery can be affected by the time interval of functional tests and exercise. For instance, the beneficial effect of BRJ supplementation on MVIC recovery was observed only at 72 h after exercise [36], while at intervals of 30 min, 24 h, and 48 h after exercise, BRJ supplementation had no effect compared to PLA [36]. In addition, the improvement in VJH after BRJ supplementation was also observed at intervals of 24, 48, and 72 h after exercise [36]. However, the heterogeneity of the reviewed results has been reported to be 67% or more [36], suggesting a large variability of the data and differences from the findings of the reviewed studies. In addition, some studies have found no positive effect of BRJ supplementation compared to PLA for VJH performance 48 h after the exercise test [24,32]. Additionally, most studies showing the positive effect of BRJ supplementation on sports performance recovery have been predominantly male [22,32,37,42], while the present study recruited female volleyball players. Therefore, the gender difference in the participants might partially explain the disparity in the results. Also, the EIMD used in this study has great biomechanical proximity and specificity to the training and game activities of the participants (volleyball). This argument is consistent with the findings by Clifford et al. (2017) on trained endurance runners [24].

Increasing the duration of the wall-sit test shows that BRJ supplementation can improve the recovery of isometric endurance performance. This function can be translatable, as volleyball players usually adopt similar positions to wall-sit or static squats to receive serves or spiking from the opponent’s team. On the other hand, Jonvik et al. (2020) showed that six days of BRJ did not significantly improve endurance performance and muscle strength in recreationally active men when compared to PLA supplementation [43]. However, the PLA supplementation used by Jonvik et al. (2020) was nitrate-depleted beet juice [43], where other BRJ nutrients may have influenced the observed results. During the present study, muscle endurance performance was measured by an isometric wall-sit test, while Jonvik et al. (2020) calculated the workload resulting from 30 bilateral voluntary isokinetic contractions at a speed of 180 degrees per second [43]. Therefore, the difference in the type of sports test (static vs. dynamic) and the measurement tools can partially explain the disparity in the findings. Consistent with the results of this study, Ranchal-Sanchez et al. (2020) showed the positive effects of BRJ supplementation on improving muscle endurance in resistance training [44]. Several other studies have also shown the positive effects of BRJ supplementation on muscle endurance [12,42,45].

The present study found that Sw-T and muscle soreness decreased significantly after BRJ supplementation. One possible explanation for the significant improvement in wall-sit performance and non-significant VSFT is the reduction in muscle soreness and related mechanisms, such as the reduction in tissue swelling [38,46]. In fact, muscle soreness is associated with symptoms such as reduced muscle force production, pain, mechanical damage to skeletal muscles, and tissue edema [38,46,47]. Furthermore, nitrate-rich dietary supplements, such as BRJ, have been reported to play a vital role in improving neuromuscular efficiency, especially during fatigue [48]. Therefore, the improvement in neuromuscular efficiency can partially explain the improved performance in the wall-sit test. Moreover, the vasodilatory effects of NO may positively influence muscle endurance performance by increasing blood flow and, in turn, providing more oxygen to muscle tissue [44]. Of note, this increased NO can also cause hypotension and/or blood redistribution away from skeletal muscle [39]. Therefore, manipulating NO production in skeletal muscles may not always lead to increased performance [39]. Furthermore, NO can induce pain perceptions after eccentric exercise. NO inhibitors may lead to a decrease in blood flow in skeletal muscles and, consequently, a reduction in muscle function [39,40]. Therefore, according to Radak et al. (2012), “you may have to pay a little pain to increase your endurance capacity” [39]. However, specific stimuli for increasing muscle NO can potentially increase blood flow to muscle tissue during exercise, repair muscle damage, and improve performance by activating satellite cells and increasing follistatin expression [39]. Hence, more studies are needed to elucidate the interaction between NO and neuromuscular efficiency post-BRJ supplementation. In addition to stimulating NO, BRJ is a source of various carbohydrate compounds (glucose), vitamins, and minerals. Therefore, BRJ can play a role in restoring muscle glycogen stores, improving immune function, and reducing inflammation due to exercise [49]. Hence, the improvement in endurance performance in the present study may also be related to other compounds in BRJ. Future studies should assess the markers of immune, metabolic, and hormonal function to establish additional mechanisms for the effects of BRJ on performance recovery. In addition, gastrointestinal complications caused by BRJ can limit its use as a comprehensive nutritional supplement in athletes. Additional studies should be conducted to reduce or prevent complications. Coaches and sports nutritionists should verify BRJ sensitivity before its prescription.

The present study has some limitations, such as the lack of measurement of the markers related to muscle damage. In addition, cognitive indicators, arousal, and other psychological variables affecting muscle function were not evaluated in the present study. Therefore, future studies should include the evaluation of these variables to expand on our findings. Moreover, some repeated bout effects may have confounded the results of the present study. Furthermore, it is important to note that the sample size used in this research was limited due to the nature of a complete volleyball team, which typically consists of 12 to 14 players. Consequently, the findings obtained from this study should be approached with careful consideration and interpreted with caution.

## 5. Conclusions

Overall, this study showed that BRJ supplementation in female volleyball players improves static muscular endurance performance 48 h after EIMD, which is also associated with reducing the perception of muscle soreness and tissue edema. Female volleyball players may use BRJ supplements after matches or high-pressure training for improved recovery of muscle endurance and pain reduction. However, PPT, VJH, and lower body flexibility did not change compared to PLA. Therefore, more studies on the timing, dosage, and manipulation of BRJ compounds and their effects on the recovery of performance indicators and muscle soreness should be conducted on female athletes in order to expand on our outcomes.

## Figures and Tables

**Table 1:** Demographic information of the participants.

Variable	Mean ± SD
Age (years)	26.00 ± 3.00
Height (cm)	174.08 ± 3.94
Body mass (kg)	67.75 ± 5.14
Body Mass Index (kg/m^2^)	22.3 ± 2.5

**Table 2:** Nutritional details of BRJ and PLA (estimated) drinks.

	BRJ	PLA
Volume (mL)	400	400
Energy (Cal)	183	180
Carbohydrate (g)	40	45
Sugar (g)	36	2.5
Fat (g)	0	0
Protein (g)	5.6	0.9
Nitrate (mmol/L)	4.1	<0.5

**Table 3:** Comparison of muscle soreness, swelling, and functional test variables in BRJ and PLA conditions.

95% CI	Sig	MD	RC %	BRJ	PLA	Variable
				Mean ± SD	Mean ± SD	
−1.2 to 1.4	0.9	0.1	−1.22%	8.1 ± 1.7	8.2 ± 1.7	MS baseline (mm)
0.4 to 19.4	0.04	9.9	−19.8%	40.2 ± 7.9	50.1 ± 12.1	MS-12 h (mm)
5.8 to 24.8	0.004	15.3	−34.1%	29.8 ± 10.3	45.2 ± 8.7	MS-24 h (mm)
14.3 to 36.8	<0.0001	25.6	−46.2%	29.8 ± 11.9 *	55.4 ± 8.1	MS-48 h (mm)
−10.7 to 9.4	0.9	0.7	0.5%	132.7 ± 7.7	132.0 ± 11.9	PPT baseline (mmHg)
−30.0 to 9.8	0.3	10.1	20.5%	59.4 ± 45.3	49.3 ± 22.8	PPT 48 h (mmHg)
−8.3 to 9.9	0.8	0.8	−0.9%	89.1 ± 20.7	89.9 ± 19.8	Wall-sit baseline (s)
−30.3 to −6.3	0.006	18.3	29.6%	80.1 ± 30.5 *	61.8 ± 21.5	Wall-sit 48 h (s)
−2.6 to 2.4	0.9	0.0	0.0%	34.3 ± 8.7	34.3 ± 9.8	VSFT baseline (cm)
−3.3 to 0.8	0.1	1.5	4.5%	32.6 ± 10.2	31.3 ± 11.3	VSFT 48 h (cm)
−0.5 to 2.5	0.2	1.0	−3.0%	32.4 ± 4.7	33.4 ± 6.1	VJH baseline (cm)
−1.2 to 1.2	0.9	0.0	0.0%	30.0 ± 5.5	30.0 ± 6.0	VJH 48 h (cm)
−0.1 to 2.2	0.06	1.1	−1.9%	57.7 ± 4.3	58.8 ± 3.6	Sw-T baseline (cm)
0.7 to 1.5	<0.0001	1	−1.6%	60.3 ± 4.3 *	61.3 ± 4.2	Sw-T (cm)

* Significant difference between PLA and CON, CI: confidence interval; RC: Relative Change [(BRJ − PLA)/PLA]; BRJ: Beetroot juice; PLA: placebo; MS: muscle soreness; Sw-T: swelling around the thigh; PPT: pressure pain threshold; VSFT: V-Sit reach flexibility test; VJH: vertical jump height.

## Data Availability

Data sharing is applicable.
